# One size does not fit all: Qualitative interviews with individuals with overweight or obesity and healthcare providers on navigating and supporting weight management

**DOI:** 10.1016/j.obpill.2026.100262

**Published:** 2026-04-01

**Authors:** Fatima Cody Stanford, Donna Mojdami, Marie Louise Edwards, Urvi Desai, Anthony Hasan, Ashley Holub, Alice Qu, Matthew Mattera, Natalia Ankerholz, Ellen Sears, Noam Kirson, Shraddha Shinde

**Affiliations:** a Department of Medicine-Division of Endocrinology-Neuroendocrine, Department of Pediatrics-Division of Endocrinology, Boston Nutrition Obesity Research Center, 50 Staniford Street, Boston, MA, 02114, USA; b Harvard Medical School, 25 Shattuck St, Boston, MA, 02115, USA; c Eli Lilly and Company, 1500 South Harding St, Indianapolis, IN, 46221, USA; d Analysis Group, Inc, 111 Huntington Ave, Boston, MA, 02199-7668, USA

**Keywords:** Obesity, Obesity medication, Overweight, Weight management

## Abstract

**Background:**

Obesity and overweight are significant health concerns in the United States (US) with an expanding number of treatment options. Patients face challenges of a complexity of options, systemic access barriers, and need for support from healthcare providers (HCPs). The goal of this study was to qualitatively assess the experience of individuals with overweight or obesity and HCPs navigating weight management, strategies including obesity medications (OMs), and the support and motivation needed for sustained success.

**Methods:**

This cross-sectional qualitative study included adults with overweight or obesity with a desire to lose weight (n = 40) and HCPs (n = 25) in the United States. Trained moderators conducted in-depth one-on-one interviews in English or Spanish. Four independent coders analyzed transcripts for each group (Cohen's kappa>0.7 for each). Narrative themes were developed summarizing key findings.

**Results:**

Four key themes were formed: (1) Participants felt overweight and obesity should be treated like other chronic diseases (individuals with overweight or obesity: 88%; HCPs: 92%). (2) Approaches to managing and discussing weight with HCPs are highly individualized. Individuals most commonly used diet (81%), exercise (61%), and OMs (28%). HCPs prioritized diet and physical activity. All HCPs recommended OMs for some patients but noted systemic access barriers such as limited insurance coverage. Individuals wanted more information on OMs, especially safety and tolerability, despite most HCPs (88%) reporting sharing this information. (3) Supporting and motivating individuals through their weight-loss journey is critical; HCP support is also critical. (4) Modifying systemic barriers could aid weight loss, including more proactive HCP discussions, more education, and improved OM access.

**Conclusion:**

This qualitative study demonstrated that approaches to managing and discussing weight are highly individualized. Support and motivation are crucial to maintain long-term success. Access to treatments and HCP support represent key opportunities for improvement for individuals seeking to achieve sustained weight loss.

## Graphical abstract

**Figure:**
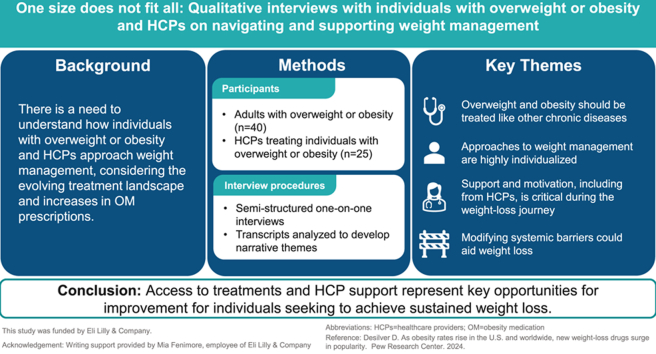

## 1 Introduction

Overweight and obesity are significant health concerns, with overweight impacting 31% and obesity impacting 43% of adults in the United States (US) [1,2]. Obesity is a complex disease impacting physical and mental health, quality of life, and social outcomes [3,4]. It is associated with comorbid conditions such as diabetes, cardiovascular disease, obstructive sleep apnea, osteoarthritis, and depression [5]. Additionally, individuals with overweight or obesity often experience stigma, bias, and discrimination, which can further affect health and economic outcomes and social relationships, and may even hinder their ability to seek treatment [[6], [7], [8], [9], [10], [11], [12], [13], [14], [15]].

Approaches to reducing weight include behavior-based interventions emphasizing lifestyle changes, and in more severe cases, metabolic and bariatric surgery [16,17]. Obesity medications (OMs), including highly effective incretin-based therapies semaglutide and tirzepatide [18,19], have expanded options for individuals with overweight or obesity to safely and effectively lose weight and reduce weight-related comorbidities [20]. At the same time, responses to these therapies may vary, and some individuals may experience adverse events or challenges with tolerability that may impact treatment continuation.

As newer treatments become available, it may be challenging for individuals to navigate the myriad of weight management options to find sustainable strategies suitable for their unique circumstances. Individuals may try multiple approaches to manage their weight over time and through stages of life, often influenced by family, friends, broader societal trends, and media [21]. Further, some individuals' options are limited by systemic barriers, including lack of healthcare access, lack of insurance coverage, stigma, discrimination, and socioeconomic status, among others [[6], [7], [8], [9],[22], [23], [24], [25], [26], [27], [28], [29], [30], [31], [32], [33], [34]]. These constraints are exacerbated among underserved and vulnerable communities, which are also highly impacted by obesity [[22], [23], [24],^27^,^29^,[31], [32], [33], [34], [35]].

Healthcare providers (HCPs) can give critical support in identifying safe and effective weight management strategies. With the addition of OMs, HCP guidance is paramount to help patients understand OM administration, benefits and risks, eligibility for medication insurance coverage, and whether an OM may be clinically appropriate and accessible given limited insurance coverage, costs, and limited supply. However, discussions about weight management options and specialist referrals may be constrained by limited consultation time, weight bias, prioritization of other health conditions, limited HCP knowledge, and HCPs’ perceived lack of patient compliance and motivation [6,8,24,[36], [37], [38], [39], [40], [41], [42], [43], [44], [45], [46], [47], [48], [49]].

The experience of navigating treatment options such as physical activity, diet, and metabolic and bariatric surgery has been well-explored [21,39,[50], [51], [52], [53]]. While some studies have considered OM use [36,[54], [55], [56], [57], [58]], there is limited in-depth understanding of the experience of an expanded, dynamic treatment landscape with rapid increases in OM prescriptions [59]. Further evidence is important for understanding how weight management options, including OMs, are considered and discussed, and for identifying ways to better support personalized, sustainable weight management.

### 1.1 Study purpose

To address these gaps in the era of new OMs, we conducted semi-structured interviews with adults with overweight or obesity who expressed a desire to lose weight and HCPs who treat obesity and overweight, aiming to better understand the real-world experience of navigating weight management options and how to best motivate and support individuals.

## 2 Methods

### 2.1 Study design and participants

For this cross-sectional qualitative study, we conducted one-on-one semi-structured interviews with 40 individuals with overweight or obesity and 25 HCPs treating individuals with these conditions in the US.

The sample size was derived based on literature review, medical expert consultation, and previous studies which have suggested that identifying the majority of commonly occurring concepts may be achieved with as little as 15 interviews [60].

Adults aged ≥18 meeting Food and Drug Administration OM indicated use criteria, defined as obesity (body mass index [BMI] ≥30 kg/m^2^ based on self-reported weight and height); or overweight (BMI 27 kg/m^2^ – < 30 kg/m^2^ with ≥1 weight-related comorbid condition [e.g., hypertension, dyslipidemia, cardiovascular disease, obstructive sleep apnea, Type 2 diabetes]) were eligible for the study. For Asian or Native Hawaiian/Other Pacific Islander individuals, we applied lower BMI thresholds for obesity (BMI ≥30 kg/m^2^) and overweight (BMI ≥23 kg/m^2^ – < 25 kg/m^2^ with ≥1 weight-related comorbid condition) to account for known heterogeneity in body composition and cardiometabolic risk across racial and ethnic groups, and to reduce potential under-recognition of adiposity-related risk when using standard BMI cut points alone [[61], [62], [63]]. We applied recruitment quotas based on participant age at screening so the study population would be evenly distributed across ages 18–39, 40–64, and ≥65. Recruitment procedures also considered the racial/ethnic distribution of our sample, aligning it with those most impacted by overweight and obesity in the US based on Centers for Disease Control and Prevention estimates [64], such that: 1) 40% identified as Non-Hispanic Black or African American, American Indian, or Alaska Native; 2) 30% identified as Hispanic, 3) 20% identified as Non-Hispanic White; 4) 10% identified as Non-Hispanic Asian, Native Hawaiian or Other Pacific Islander. Participants who identified with more than one race/ethnicity were classified into the group with the larger sample requirement. For example, a participant selecting both “Non-Hispanic/Latino White or Caucasian” and “Non-Hispanic Black or African American” would be in the “Non-Hispanic Black or African American, American Indian, or Alaska Native” group.

Eligible HCPs were board-eligible/board-certified physicians or other clinicians who prescribe medications (i.e., physician associate/physician assistant [PA], nurse practitioner) for treating individuals with overweight or obesity. Recruitment quotas were applied based on clinical degree, such that 1–2 non-physician clinicians were included, and medical specialty, with even representation from each category: 1) obesity medicine or metabolic and bariatric surgery; 2) endocrinology; 3) gastroenterology or cardiology; 4) primary care.

The study was exempted from institutional review by Pearl Independent Review Board, Indianapolis, Indiana.

### 2.2 Study procedures

We recruited participants from market research panels maintained by M3 Global Research. M3 contacted panel members to assess their interest in participating in the study. Interested panel members completed an online screener to determine eligibility and self-report demographic and clinical characteristics. Eligible participants provided consent to participate in the study and were compensated for their participation.

Each of the 65 participants meeting the selection criteria participated in a one-on-one, semi-structured, virtual interview lasting approximately 45–60 min. Interviews were conducted September–October 2023 by trained professional moderators based on semi-structured interview guides developed from a comprehensive literature review and discussions with clinical experts, including Dr. Stanford. For individuals with overweight or obesity, interview topics included their perception of and experience living with overweight or obesity, key factors influencing their weight management, perceptions of and experience with treatment options including OMs, experience with HCPs discussing weight management and referrals, and weight management support (e.g., from HCPs, family members, friends, online communities). For HCPs, interview topics included their perception of overweight and obesity, experience treating individuals with overweight or obesity, discussions about weight management strategies, recommendations and perceptions of treatment options and referrals, and how to evaluate success and motivate weight management.

Interviews were conducted in English for HCPs and English or Spanish for individuals with overweight or obesity. Interviews were audio-recorded, transcribed, and de-identified for coding. Spanish interviews were translated into English transcripts.

### 2.3 Analysis

We used SAS, version 9.4 (SAS Institute Inc., Cary, NC, USA), Microsoft Excel, and NVivo 12 Plus software to analyze the study data. We summarized participant characteristics for individuals with overweight or obesity and HCPs using descriptive statistics. For the interview portion, we used a hybrid deductive and inductive coding approach over two phases to identify concepts and sub-concepts reported by each group and developed key narrative themes across both interview sets [65,66]; additional details are provided in Supplemental Appendix A. We stratified selected concepts and sub-concepts based on participant characteristics (individuals with overweight or obesity: age, BMI, race/ethnicity, income, and education level (a proxy for health literacy); HCPs: medical specialty). Given the small sample sizes of the stratifications, stratified analyses were conducted for informational purposes only.

## 3 Results

### 3.1 Participant characteristics

#### 3.1.1 Individuals with overweight or obesity

The study included 40 individuals with overweight or obesity (Table 1). See Supplemental Appendix A and Supplemental Fig. 1 for information on recruitment and enrollment. Thirty (75%) participants were interviewed in English and 10 (25%) in Spanish (denoted with “M” in the participant ID). Most participants were female (60%). Age (mean ± standard deviation [SD]: 51.9 ± 14.0 years) was evenly distributed across age groups (18–39, 40–64, and 65+) per study design. Race and ethnicity distribution followed study design, with most identifying as non-Hispanic Black or African American, American Indian, or Alaska Native (40%) or Hispanic (30%). Most lived in the South (50%) or Midwest (23%). Over half of the population had a college or graduate degree (53%), and 40% had some college or an Associate's degree. All were insured; most were covered by private (50%) or Medicare (40%) insurance.

**Table 1:** Characteristics of individuals with overweight or obesity^a^.

	Total (N = 40)
**Demographics**	
Age (years)	
Mean ± SD	51.93 ± 13.96
Gender, N (%)	
Female	24 (60.0%)
Male	15 (37.5%)
Non-binary	1 (2.5%)
Race/ethnicity^b^, N (%)	
Non-Hispanic/Latino Black or African American	13 (32.5%)
Non-Hispanic/Latino White or Caucasian	10 (25.0%)
Hispanic or Latino	10 (25.0%)
Asian	4 (10.0%)
American Indian or Alaska Native	3 (7.5%)
Hispanic/Latino White or Caucasian	2 (5.0%)
Geographic region, N (%)	
South	20 (50.0%)
Midwest	9 (22.5%)
Northeast	7 (17.5%)
West	4 (10.0%)
Highest level of education completed, N (%)	
4-year college degree or equivalent	11 (27.5%)
Graduate or doctorate degree or equivalent	10 (25.0%)
Some college	9 (22.5%)
Associate's/2-year degree or equivalent	7 (17.5%)
High school graduate or GED/high school equivalency	2 (5.0%)
Vocational/trade school	1 (2.5%)
Employment status, N (%)	
Working, full-time	15 (37.5%)
Retired	14 (35.0%)
Working, part-time	7 (17.5%)
Unemployed, not looking for work	2 (5.0%)
Other^c^	2 (5.0%)
Household annual income, N (%)	
Less than $15,000	1 (2.5%)
$15,000 to less than $30,000	11 (27.5%)
$30,000 to less than $50,000	6 (15.0%)
$50,000 to less than $75,000	9 (22.5%)
$75,000 to less than $100,000	8 (20.0%)
$100,000 or greater	5 (12.5%)
Current primary insurance, N (%)	
Private	20 (50.0%)
Medicare	16 (40.0%)
Medicaid	3 (7.5%)
Tricare/VA health care	1 (2.5%)
**Clinical characteristics**	
Body mass index^d^	
Mean ± SD	37.66 ± 6.46
Waist circumference (inches)	
Mean ± SD	44.43 ± 6.22
Missing, N (%)	4 (10.0%)
Obesity/overweight status^e^, N (%)	
Overweight (BMI 27 kg/m^2^ – < 30 kg/m^2^)	3 (7.5%)
Class 1 obesity (BMI 30 kg/m^2^ – < 35 kg/m^2^)	12 (30.0%)
Class 2 obesity (BMI 35 kg/m^2^ – < 40 kg/m^2^)	10 (25.0%)
Class 3 obesity (BMI ≥40 kg/m^2^)	15 (37.5%)
Weight-related comorbidities^b^, N (%)	
High blood pressure or hypertension	23 (57.5%)
High cholesterol or hyperlipidemia	20 (50.0%)
Type 2 diabetes	16 (40.0%)
Obstructive sleep apnea	14 (35.0%)
Osteoarthritis	13 (32.5%)
Cardiovascular disease	2 (5.0%)
Other health condition	2 (5.0%)
None	3 (7.5%)
**Treatment experience**	
Experience with OMs, N (%)	
Never used	28 (70.0%)
Prior use	8 (20.0%)
Currently using	4 (10.0%)
Experience with metabolic or bariatric surgery, N (%)	
No prior or planned metabolic or bariatric surgery	35 (87.5%)
Had prior metabolic or bariatric surgery	5 (12.5%)
Scheduled or upcoming metabolic or bariatric surgery	0 (0.0%)

**Abbreviations**: BMI = body mass index; GED = General Educational Development; kg = kilogram; m = meter; OMs = obesity medications; SD = standard deviation; VA = Veterans Affairs.

**Notes**:

a All characteristics were described at the time of the screener.

b Multiple responses were allowed, so counts and percentages may not sum to the total N or 100%.

c 1 person listed employment status as “disabled/homemaker” and 1 person listed employment status as “disabled".

d BMI was calculated based on self-reported weight and height as weight (lb)/[height (in)]^2^ ∗ 703.

e For Asian or Native Hawaiian/Other Pacific Islander individuals overweight/obesity condition was defined as: 23 kg/m^2^ - < 25 kg/m^2^ (overweight)- 25 kg/m^2^ - < 30 kg/m^2^ (Class 1 obesity)- 30 kg/m^2^ - < 35 kg/m^2^ (Class 2 obesity)- ≥ 35 kg/m^2^ (Class 3 obesity).

The individuals with overweight or obesity had a mean BMI of 37.7 ± 6.5 kg/m^2^ and mean waist circumference of 44.4 ± 6.2 inches. Over 90% had obesity (Class 1 obesity (BMI 30 kg/m^2^ – < 35 kg/m^2^): 30%; Class 2 obesity (BMI 35 kg/m^2^ – < 40 kg/m^2^): 25%; Class 3 obesity (BMI ≥40 kg/m^2^): 38%). The most common weight-related comorbidities were hypertension (58%), high cholesterol or hyperlipidemia (50%), and Type 2 diabetes (40%). At the time of screening, 10% reported using OM, and 20% reported previous OM use. Five (13%) participants reported history of metabolic or bariatric surgery (Table 1).

#### 3.1.2 HCPs treating patients with overweight or obesity

The study included 25 HCPs treating patients with overweight or obesity (Table 2). See Supplemental Appendix A and Supplemental Fig. 1 for information on recruitment and enrollment. HCPs were predominately male (76%), aged 35–44 (48%) or 45–54 years (36%), and identified as Asian (44%), Non-Hispanic/Latino White or Caucasian (28%), and Hispanic/Latino White or Caucasian (12%). The specialty mix aligned with study design: primary care providers (PCPs), including one PA (28%), endocrinologists (24%), metabolic and bariatric surgery providers (24%), gastroenterologists (20%), and cardiologists (4%). HCPs had an average of 12.3 ± 6.6 years in practice. Most practiced in the South (52%). Practice settings included private practice offices or clinics (48%), academic institution or university medical center hospitals or clinics (40%), non-academic hospitals or hospital-owned clinics (not VA or government) (28%), and community practice (20%), with overlap between practice settings. Over half of HCPs (52%) stated that individuals with overweight or obesity represented 60% - <80% of the patients they treat. HCPs most commonly treated individuals with private insurance and Medicare. All had experience prescribing OMs (Table 2).

**Table 2:** Characteristics of HCPs treating individuals with overweight/obesity^a^.

	Total (N = 25)
**Demographics**	
Age^b^, N (%)	
25–34 years old	2 (8.0%)
35–44 years old	12 (48.0%)
45–54 years old	9 (36.0%)
55–64 years old	2 (8.0%)
65 years old and older	0 (0.0%)
Gender, N (%)	
Male	19 (76.0%)
Female	6 (24.0%)
Race/ethnicity^c^, N (%)	
Asian	11 (44.0%)
Non-Hispanic/Latino White or Caucasian	7 (28.0%)
Hispanic/Latino White or Caucasian	3 (12.0%)
Hispanic/Latino Black or African American	1 (4.0%)
Non-Hispanic/Latino Black or African American	1 (4.0%)
Declined to answer	2 (8.0%)
**Clinical experience**	
Medical specialty^d^, N (%)	
Primary care	7 (28.0%)
Endocrinology	6 (24.0%)
Metabolic and bariatric surgery care	6 (24.0%)
Gastroenterology	5 (20.0%)
Cardiology	1 (4.0%)
Number of years in practice (years)	
Mean ± SD	12.32 ± 6.55
**Practice characteristics**	
Geographic region, N (%)	
South	13 (52.0%)
Midwest	5 (20.0%)
Northeast	5 (20.0%)
West	2 (8.0%)
Practice facility type^d^, N (%)	
Private practice office or clinic	12 (48.0%)
Academic institution or university medical center hospital or clinic	10 (40.0%)
Non-academic hospital or hospital-owned clinic (not Veteran's Affairs or government)	7 (28.0%)
Community practice	5 (20.0%)
Percentage of patients with obesity or overweight^e^, N (%)	
20% to less than 40%	1 (4.0%)
40% to less than 60%	6 (24.0%)
60% to less than 80%	13 (52.0%)
80% or more	5 (20.0%)

**Abbreviations**: HCP = healthcare provider; SD = standard deviation.

Notes:

a All characteristics were described at the time of the screener.

b The screener collected age ranges.

c Multiple responses were allowed, so counts and percentages may not sum to the total N or 100%.

d 1 physician associate/physician assistant was recruited and specializes in primary care (family medicine).

e For Asian or Native Hawaiian/Other Pacific Islander individuals overweight/obesity condition was defined as: 23 kg/m^2^ - < 25 kg/m^2^ (overweight)- 25 kg/m^2^ - < 30 kg/m^2^ (mild obesity)- 30 kg/m^2^ - < 35 kg/m^2^ (moderate obesity)- ≥ 35 kg/m^2^ (severe obesity).Else, it was defined as: 27 kg/m^2^ - 30 kg/m^2^ (overweight)- 30 kg/m^2^ - < 35 kg/m^2^ (mild obesity)- 35 kg/m^2^ - < 40 kg/m^2^ (moderate obesity)- ≥ 40 kg/m^2^ (severe obesity).

### 3.2 Interview results

Below, we summarize the findings for key themes discussed in interviews with individuals with overweight or obesity and HCPs. Table 3 lists quotes supporting these themes. Upon completion of interviews, concept saturation was achieved as no new information was being reported by interviewees.

**Table 3:** Quotes supporting key themes.

Themes	Sub-category	Patient/HCP quotes^a^^,^^b^
**Theme 1: Overweight or obesity should be treated like other diseases. It is complex and multifactorial, with profound impacts on health and life.**		*[Patient 114] Well, my experiences of being overweight is that people do not see you as a person; they see you with weight, and then people judge you because of your weight. And it is not fair.*
		***[Bariatric Surgeon 96]****I think obesity is a disease like patients who have high blood pressure or diabetes. It needs to be treated as such. It's [caused by] a combination of factors. I don't think there's one individual factor that makes patients obese.*
		*[Patient 72] [Obesity] messed with my mobility, my ability to move around. And it messed with my ability to function on a daily basis. [ …] It really affects your self-esteem as well. It makes you feel really self-conscious a lot …*
**Theme 2: Approaches to weight management and discussions about weight are highly individualized**	Definitions of success in weight management	*[Patient 20] I really want to be under 200 [pounds]. So, I do have a weight loss goal because of how I think it's going to affect my knees, specifically. I will talk about some of my successes. I've gone off blood pressure medicine. I've gone off insulin. So, I'm trending the way I want to be trending. I just want to go a little bit farther.*
		*[Patient 14] I would like to lose enough weight to where I can get up and do things with my family and enjoy activities with my grandchildren, and just comfortably be able to breathe good enough to go do those things.*
		***[PCP 86]****I think my success is my patients have lost good amount of weight. I can put a number. I would say at least 10 to 15% of body weight. That is my success. And patient is consistently able to maintain that weight and change their lifestyle. I think that is a success story.*
		***[Endocrinologist 2]****[I measure success by] looking at the numbers of the weight itself, but also looking at the comorbidities … looking at the glycemic control, looking at their blood pressure. […] Are they reducing the number of medications they're taking, for example, if the controls become improved? This [improvement in comorbidities] has improved the quality of life now.*
	Strategies currently pursued by individuals with overweight or obesity and recommended by HCPs	*[Patient 13] [I use] [j]ust diet control. I've tried to do some exercising cautiously. I've been trying to manage or balance the two of them.*
		*[Patient 148] [I am] [m]aking lifestyle changes [to lose weight], I tend to really follow social media a lot. Any videos or seeing what other people switch their meals to or recipes or things like that, I'll follow those and do ones that suit my routine better.*
		*[Patient 10] Well, I've seen some shots [AOMs] that are advertised on television. That looks interesting. I know there are some weight loss pills that I could take. But at the same time, I'm always afraid of side effects.*
		*[Patient M2] I do not consider medications because I feel that you would have to take them for your entire life.*
		*[Patient 150] I've actually been looking into [surgery]. I have a couple of people that I am acquainted with … who have gone through the lap band surgery …. They've lost significant amount of weight. There are also some people … who have lost the weight and then put it back on massive. I've just been trying to weigh my options just through their experiences and just through my own personal research, as well.*
		***[PCP 63]****I might talk about [anti-obesity] medications [to my patients] and … see how we can make some compromises on the diet. The lifestyle, the exercise, I feel like everybody should be doing because there is additional benefits from exercise just beyond losing weight and controlling obesity.*
		***[Bariatric Surgeon 103]****Well, [I use] all of the options [of weight management strategies] available. Number one is dietary modification for everybody, regardless, right? Number two, everybody gets evaluation by health coach/exercise physiologist. Everybody get an evaluation by psychologist to see if there is any underlying eating disorder or maybe some untreated psychological condition, maybe anxiety, maybe depression … [Then,] they are prescribed with weight-loss medications. We offer intragastric balloon treatment for people who qualify. We offer bariatric surgery.*
		***[PA 11]****Sometimes, [the treatment consideration] it's just the patient's interest. Sometimes, it's the patient's insurance. Sometimes, it's their comorbidities. Sometimes, it's their age. Sometimes, it's whether they have a needle phobia, so different things.*
	Discussions with HCPs about weight management	*[Patient M4] He [my doctor] started it [the weight management discussion] because he saw that I was not losing weight and my blood and glucose levels were not lowering either. So, he said, we have to put a plan into action. But he also saw the need, when I expressed my need to also want to address the psychological issue. So it was like a conversation that he said, well, we're going to approach it from 360°, not just one side, but we're going to try to put the two things together.*
		*[Patient 75] We [my doctor and I] basically discussed how I'm doing, if I feel like I've lost any weight, what am I doing, am I moving more and stuff, but she's just asking if I'm keeping up with the exercise. She just touches briefly on it. But usually, it's me that brings it up.*
		***[Gastroenterologist 4]****Sure. I mean, it [barriers to discussing weight management] could be a lot of things. It could be time. I mean, if I've got other primary concerns, I may not just have a chance to get to the obesity part.*
		***[Bariatric Surgeon 96]****I feel pretty comfortable talking to all my patients [about] weight of management. Patients who had bad experiences, they're a little bit more reluctant to new approaches and whatnot. Nowadays, patients are very well informed, internet and social media, friends and family, obviously, but mostly social media plays a significant role. They have a better understanding of their disease process and they already researched.*
**Theme 3: Supporting and motivating individuals with overweight or obesity through their weight-loss journey is critical**	Support from friends and family	*[Patient 159] I have support from friends and family, they tell me that they're proud of me. Just hearing those words of affirmation, things like just being able to fit smaller sizes. So those are some of the things that are really positive for me.*
		*[Patient 22] I have a huge online presence in terms of weight loss groups, coaches and things like that. I absolutely have that kind of support. I have a lot of friends in the weight loss management community, who I talk to on a daily basis, and we support each other.*
	Support from HCPs	*[Patient 72] It would be nice if I could get some support from … the two doctors that keep mentioning my weight, it would be nice if they could help me as far as what exactly would help me, instead of just saying you are overweight.*
		*[Patient 116] [I expect my doctor] [j]ust to support my efforts, to hold me accountable. I don't know what I really what more I would expect from him [my doctor]. He's supportive. He would like to see me do well ….He is a good listener. He is very empathetic. That's it. I don't really know what else I'd want from him.*
		***[Bariatric surgeon 35]****I tell them to lose weight and I keep telling them, “Hey, by cutting down your weight, you'll see what happened to your blood pressure. Now you need only three that we cut down and take you off this one medication.” That alone can be a positive reinforcement.*
		***[Endocrinologist 89]****I give them positive reinforcement. When I do notice that they've lost a significant amount of weight, and I congratulate them. I ask them to tell me how they did it, the kinds of changes that they made. […] I show them how it's– having a positive impact on their blood work, on the other parameters that we're measuring. […] I try not to provide negative reinforcement if the patient has not lost much weight. I try to find out, What do you think is the barrier that's getting in your way?*
	Motivation to use OMs	***[Gastroenterologist 35]****I would say the one [reason for not filling an AOM prescription is] most likely there won't be insurance or their copay. Other ones would be, they have started, they initially filled, now they're having some side effect. That would be the other one.*
		***[PCP 57]****Well, it [using AOMs] typically does not require too much motivation. … [I]f we have reached that point, it typically means that patients also want to do it. The only extra thing that I can do to help that is it would be providing samples or be providing extra assistance needed or maybe getting it approved from the insurance companies.*
**Theme 4: There is still room for improvement: Individuals with overweight or obesity and HCPs highlighted systemic barriers that could be modified to help patients achieve success and improve treatment satisfaction**	Unmet needs in weight management discussions	*[Patient 10] Like I've said, I feel odd [about bringing up my weight to my doctor]. I feel ashamed. If it's that big of a deal, is that big a problem, they should be mentioning it.*
		*[Patient 78] My current physician is [not offering me support], other than just the quick check-the-box conversation. But what I would want to see [is my doctor] … taking a little more time not making me feel like she's rushed to go to the next appointment, to actually have a conversation.*
	Unmet needs in OM education and access	*[Patient 39] Well, I didn't know about these drugs [AOMs]. So, if he [my doctor] said something about these drugs to me and what their side effects were and things like that, that could be useful information.*
		*[Patient 75] [I would like] [j]ust more knowledge as far as options, what qualifies you for different kind of, I don't know, medicines or therapies.[…]Knowing what's out there, and what I would qualify for, I think, would be so helpful. Because the only thing I know is what I read online or if I go to my doctor with questions, or something like that.*
		***[Endocrinologist 2]****More [educational] resources for patients would be helpful. … [M]ore resources that make it easier for the providers[…]. Patient education material will be helpful as we get more and more options, [and] … help providers understand which patients will be better for which sorts of options.*
		***[Bariatric Surgeon 103]****Well, I think more education is needed to the general public, to educate the general public that obesity is a disease and that there are treatment options available, and they shouldn't be shy on seeking obesity treatment.*
		*[Patient M1] [Are finances a factor that stops you?] Very much so. I did check it, and bariatric surgery is about 5000 or 6000. The injections [AOMs] they offered me for 90 days were 300 USD … Then I thought if I invest in this and the injections do not change me? Then I get cold feet, and I do not do anything.*
		*[Patient 13] My doctor tried to give me a coupon where I could get it for $25 a shot. I took it to the pharmacy. The pharmacist said, “I'm sorry, this coupon doesn't apply to Medicare patients.” I said, “Those are the people that need it.” Medications are out there, but I cannot access them.*
		***[Gastroenterologist 33]****I guess [an improvement could be in] the medications, GLP-1 agonist appears to be effective for the vast majority of patients. The problem I'm having is insurance companies are not covering it. It's primarily an issue of insurance access.*

**Abbreviations**: AOMs = anti-obesity medications; HCP = healthcare provider; OMs = obesity medications.

Notes:

a Identifiers for quotes from HCPs bolded to distinguish from quotes from individuals with obesity or overweight (identified as “patients”).

b Patient/HCP quotes reference the term AOM, which is otherwise referred to as OM in the text and table.

#### 3.2.1 Theme 1: Overweight or obesity should be treated like other diseases. It is complex and multifactorial, with profound impacts on health and life

**Fig. 1:**
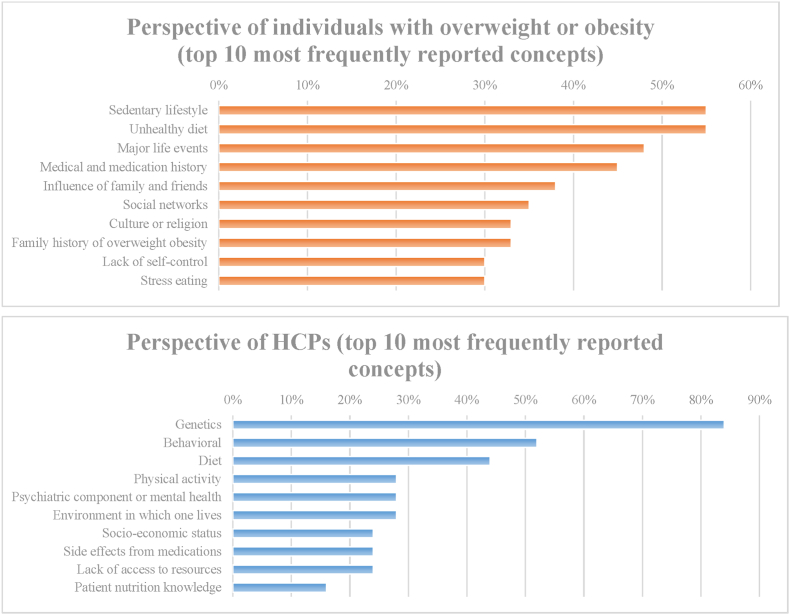
Factors contributing to overweight and obesity^a^^-^^^c^^. **Abbreviation**: HCP = healthcare provider. **Notes**: a. Multiple responses were allowed per participant. b. Medical and medication history includes mental health, other medical conditions (e.g., chronic pain), and pregnancy. c. Access to resources includes food and healthcare options.

Obesity profoundly impacts individuals' health, well-being, and lived experience. Almost all individuals with overweight or obesity (88%) and HCPs (92%) felt that overweight and obesity are conditions that should be treated similarly to other diseases, such as diabetes and hypertension. HCPs and individuals described obesity as a chronic condition with connections to comorbidities, including cardiometabolic conditions and cancer. Interviewees emphasized effects on mental health, including depression, body dissatisfaction, low self-esteem, and social anxiety, and commonly reported obesity impeding daily activities (58%; not shown in tables). Overweight and obesity were described as complex, with contributors ranging from genetics and lifestyle to societal factors. (Fig. 1). All emphasized that obesity can be difficult to resolve or control. HCPs stressed shifting blame from the individual and focusing on treating the condition in the larger context of individuals’ metabolic health and unique contributing factors.

#### 3.2.2 Theme 2: Approaches to weight management and discussions about weight are highly individualized

##### 3.2.2.1 Definitions of success in weight management

Individuals with overweight or obesity and HCPs similarly defined successful weight management (Supplemental Table 1). Success meant sustained weight loss with maintainable lifestyle changes and improving general health and specific health concerns (e.g., joint pain). This could include needing fewer medications, having more energy, and participating in activities without discomfort. Individuals with overweight or obesity also noted the importance of weight loss and appearance, though some indicated focusing on the scale as a negative.

##### 3.2.2.2 Strategies currently pursued by individuals with overweight or obesity and recommended by HCPs

Despite a similar understanding of success, weight management strategies were highly individualized. Almost all (90%) individuals with overweight or obesity had a plan to lose weight at the time of interviews, most commonly mentioning diet (81%), exercise (61%), and OMs (28%). Some discussed a preference to lose weight “naturally” or were wary of a “radical approach” like surgery. Some options, such as OMs and surgeries, were independently researched, but individuals wanted more information from their HCPs. Weight management strategy recommendations came from HCPs, family, friends, social media, and the Internet (Table 4). Younger individuals reported social media as an information source most frequently (age 18–39: n = 8; 62%; age 40–64: n = 7; 50%; age ≥65: n = 5; 38%) while older individuals tended to look to family or friends (age 18–39: n = 5; 38%; age 40–64: n = 7; 50%; age ≥65: n = 7; 54%). Social media offered broad support, including tips, recipes, exercises, and a sense of community, with potential negatives such as perpetuating negative body image. Participants noted expense and lack of insurance coverage limited their options of surgical procedures, OMs, gym memberships, and delivery meal-kit services.

**Table 4:** Strategies currently pursued by individuals with overweight or obesity and recommended by HCPs^a^.

	Number of respondents	% of N	References^b^
**Perspective of individuals with overweight or obesity**		**N = 40**	

Current strategy to manage weight			
Has a current strategy	36	90%	148
Diet	29	81%	72
Physical activity	22	61%	33
OMs	10	28%	21
Supplements	6	17%	8
Support groups	4	11%	5
Therapy	4	11%	5
Does not have a current weight management method	4	10%	4
**Source of recommendations for weight management**			
HCP	23	58%	44
Social media	20	50%	27
Friends and family	19	48%	36
Internet	18	45%	24
Commercials or TV ads	12	30%	12
Word of mouth	10	25%	15
Articles or newspapers	3	8%	3
**Source of OM information**			
Word of mouth	13	33%	23
HCP	12	30%	20
Social media	9	23%	13
Commercials and TV ads	9	23%	10
Articles or newspapers	7	18%	7
Internet	5	13%	5
Weight loss doctor	3	8%	3

**Perspective of HCPs**		**N = 25**	

**Approach to helping patients manage their weight**			
OMs	25	100%	45
Nutritional interventions (i.e., dieting)	23	92%	36
Surgical interventions	22	88%	34
Physical activity interventions (i.e., exercise)	20	80%	31
Behavioral or psychological therapy	16	64%	18
Referral to other specialist	14	56%	20
Multidisciplinary care or coordinated care team	4	16%	4
**Patient factors that impact HCP decision-making about treatment strategy**			
Clinical characteristics	21	84%	68
Comorbidities	19	76%	35
Treatment history	14	56%	26
Lab values	5	20%	7
Severity of overweight or obesity	20	80%	38
Socio-demographics	20	80%	62
Age	13	52%	15
Cultural background	9	36%	12
SES	9	36%	14
Religion	7	28%	9
Gender	5	20%	6
Ethnicity	4	16%	6
Patient motivation	18	72%	36
Insurance factors or cost	16	64%	21
Health literacy	12	48%	20
Patient lifestyle or schedule	9	36%	11
Patient preference	6	24%	11
**Factors that influence prescription of OMs**			
Patient factors	24	96%	122
Comorbidities	23	92%	45
Bariatric surgery (past or upcoming)	21	84%	34
Patient treatment history	8	32%	14
SES	5	20%	5
Severity of overweight or obesity	5	20%	7
Age	4	16%	4
Family history	3	12%	3
Features of OMs	23	92%	52
Contraindication	10	40%	10
Route of administration	10	40%	10
Length of use	9	36%	10
Side effects	8	32%	9
Efficacy	7	28%	7
Frequency of use	6	24%	6
Insurance or cost	22	88%	41
Patient preference or understanding	20	80%	39
Patients willingness to use or preference	16	64%	24
Patient health literacy	8	32%	12
Patient expectation for medication	3	12%	3
HCP knowledge of medication	3	12%	4
Medication shortage	2	8%	2

**Abbreviations**: HCP = healthcare provider; OMs = obesity medications; SES = socioeconomic status.

**Notes**:

a Response options reported by at least three participants are shown.

b Refers to number of times mentioned by respondents.

Regarding OMs, most (83%) were aware of the medications, especially younger age groups (age 18–39: n = 13; 100%; age 40–64: n = 11; 79%; age ≥65: n = 9; 69%). OM information came from various sources, including word of mouth and HCPs (Table 4). Some thought the medications were effective and safe, while some desired more information about safety, route of administration, and affordability. PCPs were the most common prescribers among those with a prescription.

HCPs' treatment recommendations were individualized and influenced by clinical characteristics, the severity of overweight or obesity, socio-demographics, perception of the individual's motivation, and insurance factors or cost. BMI-based cutoffs for treatments or clinical guidelines specific to obesity or related conditions (e.g., diabetes, cardiac conditions) were also used. HCPs prioritized diet (92%) and exercise (80%). One HCP described lifestyle changes as the “foundation of every weight loss modality.” Most HCPs (88%) reported recommending surgical interventions to some patients (Table 4). HCPs also referred to specialists to support weight management, including nutritionists and mental health providers (each 68%), bariatric surgeons (56%), and endocrinologists (48%) (Supplemental Table 2).

All HCPs recommended OMs for some patients, noting newer OM options as “less risky” and “genuinely helpful.” Some expressed concerns about side effects, options available, efficacy, and route of administration. When HCPs consider whether to prescribe OMs, decision drivers included consideration of comorbidities (92%), insurance coverage or cost (88%), and medication shortages (8%), among others (Table 4).

Several HCPs responded to questions about OM use concerning bariatric surgery, noting OMs can be helpful pre- and post-surgery. One HCP described, “These AOMs [anti-obesity medications] are a useful adjunct to surgery preoperatively … to increase some weight loss and make them a healthier candidate for surgery. And then postoperatively to continue weight loss, maintain it, and continue improving upon the surgical results.” HCPs raised concerns regarding rebound weight gain following bariatric surgery, noting OMs may be a helpful offset.

##### 3.2.2.3 Discussions with HCPs about weight management

In selecting weight management strategies, discussions between HCPs and individuals with overweight or obesity were essential decision points. Most (83%) individuals with overweight or obesity had discussed weight management with at least one HCP in the last year, primarily with a PCP (91%).

Weight management discussions did not follow specific timelines or cadences. HCPs reported discussions occurring on the first visit (52%), every visit (45%), and milestones such as when the individual has or is at risk of developing comorbidities (76%). HCPs were also prompted to discuss weight by perceiving the patient as motivated and seeing they were having trouble losing weight. Discussion barriers included prioritizing comorbidity treatment over weight management, lack of time, perception of patients' lack of motivation, or perception of their ability to pursue weight-loss strategies because of age and finances, among others. Individuals perceived a lack of discussion on weight with HCPs as due to HCPs focusing on other health conditions (20%) or not bringing the topic up (10%). Individuals most commonly reported that HCPs initiated conversation, while HCPs reported it was a mix of the patient and themselves. (Supplemental Table 3). Some individuals indicated they expected HCPs to initiate weight management discussions. Other reasons individuals did not initiate discussions with HCPs included not wanting to seek help, feeling they can manage their weight themselves, or feeling uncomfortable. One individual said they felt “ashamed” to mention their weight, but they “are hoping the doctor brings it up. It's pretty obvious.”

HCPs' approach to weight management discussions varied depending on the patient and context. For example, HCPs may tailor information delivery to patients' prior treatment experiences, perceived health literacy, or perceived comfort discussing weight loss (Supplemental Table 3). HCPs may also discuss specialist referrals. Patients' comorbidities, the severity of obesity, HCPs’ perceptions of patient willingness, and insurance requirements (Supplemental Table 2) drive these recommendations. When time is limited, HCPs may schedule follow-ups to discuss OMs in detail, have the patient call back, or ask the patient to do research before following up. Individuals with overweight or obesity reported various outcomes of discussions, mentioning the discussion led to a referral to a dietitian or nutritionist, bariatric surgeon, or therapist (50%), was related to other health conditions (43%), was related to OMs (40%), and led to action undertaken by the individual (40%). When discussing OMs, HCPs reported sharing information on side effects, dosage, length of use, efficacy, and route of administration. Individuals with overweight or obesity emphasized desiring more information, including safety and tolerability, despite most HCPs (88%) reporting sharing this information (Supplemental Table 3).

#### 3.2.3 Theme 3: Supporting and motivating individuals with overweight or obesity through their weight-loss journey is critical

##### 3.2.3.1 Support from friends and family

Interviewees described motivation and support as essential to successful weight management. Individuals were motivated to lose weight by support from friends and family, observing weight-loss results, and feeling acknowledged by others for progress. They emphasized social support through accountability buddies, workout partners, and peer communities like online support groups and social media (Supplemental Table 4). One individual described having a friend they went to the gym with. When the friend stopped coming, they explained that “losing that support of having someone come with me contributed to me not going anymore,” indicating that consistent support throughout the weight-loss journey may be necessary to make sustained changes.

##### 3.2.3.2 Support from HCPs

Individuals also emphasized HCP support, noting it makes a “world of difference when you have a doctor that knows and understands what you're doing and supports you in it.” About one-third of individuals expected their HCP to provide weight management solutions and support. Over half of individuals (58%) had an HCP currently involved in their weight management, with individuals with Class 2 obesity or Class 3 obesity reporting this more frequently (70% and 67%, respectively) than those with lower BMI (Class 1 obesity: 42%; overweight: 33%). Individuals also reported a lack of support from at least one HCP (58%), including lack of acknowledgment of weight issues, acknowledgment of weight issues without offering solutions, or focusing on other comorbidities (Supplemental Table 4). Lack of HCP support was more commonly reported by those with lower BMI than higher (Individuals with overweight: 100%; Class 1 obesity: 75%; Class 2 obesity: 40%; Class 3 obesity: 47%).

HCPs encouraged individuals with overweight or obesity by showing improvements in terms of weight loss, lab values, or medication needs. They discussed benefits of weight loss on other health conditions, kept regular appointments, offered supportive words, and acknowledged successes. They aim to set realistic goals and sometimes provide practical solutions, such as app recommendations to support progress (Supplemental Table 4).

##### 3.2.3.3 Motivation to use OMs

HCPs motivated their patients to use OMs like they approached general weight management support, including setting realistic goals for OM effectiveness, showing improvements the individual has made, and scheduling frequent follow-ups. For example, an HCP emphasized the importance of frequent follow-ups for individuals using OMs as their first medical weight-loss intervention: “they [patients] are very anxious, they are very – sometimes curious. And most of the time, they want the results faster, so to keep them compliant, I need more close follow-ups with them.” Individuals’ responses corroborated these motivation sources, as they reported filling prescriptions because they needed additional help from medications to lose weight or previously experienced benefits from use. Once prescribed OMs, HCPs described their patients as generally self-motivated, with most complying after filling their prescriptions (Supplemental Table 4). When OM prescriptions were not filled or refilled, it was primarily due to lack of insurance coverage, tolerability, and medication shortage (Supplemental Table 5). One individual described, “I lost my job. With that, I lost my health insurance. The medicine was very expensive, so I had to look for another option that had nothing to do with that medication.”

#### 3.2.4 Theme 4: There is still room for improvement: Individuals with overweight or obesity and HCPs highlighted systemic barriers that could be modified to help patients achieve success and improve treatment satisfaction

##### 3.2.4.1 Unmet needs in weight management discussions

Participants emphasized there was room for improvement in supporting individuals’ sustained weight management. There is a need for HCPs to engage patients more readily and sooner (Supplemental Table 5). Individuals with overweight or obesity desired more direction, tangible solutions, and help setting priorities earlier in their journeys. Strategies should be individualized, reflecting that, as one HCP said, “[E]very patient is different, and every patient sees their disease or obesity problems differently.” Weight management discussions sometimes felt perfunctory rather than thoughtful, two-sided conversations. Others noted HCPs have not brought up weight despite patients wishing they would. Some individuals expressed desires for specialist referrals. One individual noted a referral to “a doctor who specializes in helping you lose weight” would be helpful to offer tangible guidance and keep them on track.

##### 3.2.4.2 Unmet needs in OM education and access

Next, more information and educational resources are needed about weight management and OMs (Supplemental Table 5). Individuals with overweight or obesity may benefit from increased accessible information and resources from both cost and health literacy standpoints. Such resources should also be provided to HCPs to stimulate further discussions with patients about options. Resources and education for broader society on the complex nature of the condition and challenges people with obesity face could also be helpful, encouraging more empathy.

Finally, despite motivation and support to use OMs, individuals with overweight or obesity and HCPs alike commonly reported access concerns (Supplemental Table 5). Lack of OM insurance coverage led to prohibitive costs, and interviewees noted a need for more availability due to shortages or stockouts, rendering certain medications seemingly unavailable.

Addressing these barriers of discussing weight, education, and OM access can help individuals with overweight or obesity manage their weight better, feel supported, and lead healthier lives.

## 4 Discussion

Overweight and obesity are highly prevalent, chronic conditions impacting individuals’ physical and mental health, often resulting in reduced quality of life and adverse health consequences. Previous literature has explored the treatment experience and barriers for individuals with overweight or obesity and HCPs [21,39,[50], [51], [52], [53]], with some studies incorporating OMs [36,[54], [55], [56], [57], [58]]. However, the treatment landscape is dynamic and has evolved since those studies were published, reflected by sharp increases in prescriptions and OM sales from 2018 to 2023 [59]. It continued to grow with the November 2023 approval of tirzepatide for weight loss, after our interviews were conducted. Therefore, there is a need to understand how individuals with overweight or obesity and HCPs navigate treatment as OMs become more common in weight management discussions and broader societal conversations. Our study examined the experiences of individuals and HCPs considering weight management approaches in this new treatment paradigm and how to support and motivate individuals to achieve sustained, individualized weight loss.

The American Medical Association (AMA) characterized obesity as a disease in 2013 [67]. Most participants in our study (individuals with overweight or obesity: 88%; HCPs: 92%) believed overweight and obesity should be treated like diseases such as diabetes and hypertension. Our findings may reflect a broader change in social attitudes consistent with the AMA designation, since previous research has shown mixed evidence on whether obesity is perceived as a disease. For example, a 2018 survey of 3008 individuals with obesity based on self-reported weight and height found that 65% considered obesity a disease [36]. Our participants’ perceptions may inform their openness to discuss the condition with HCPs and, for HCPs and some individuals with obesity, consideration for treatments such as OMs and surgery.

In our study, successful weight management was perceived as holistically improving health and making sustained changes to lose and keep weight off. Some emphasized establishing and maintaining self-control and implementing lifestyle changes. Likewise, interviewees consistently noted similar motivation sources and the value of support from loved ones, peers, and HCPs, findings consistent with prior literature [21,36,68]. For example, an interview study of 23 Black men living in the rural South reported the importance of social support and comfort in weight management programs [21]. Individuals in our interviews also mentioned the importance of social media as a source of information and social support, while others reported it perpetuated unrealistic body images and weight stigma, reflecting a complex environment to navigate.

While sharing common goals and motivations, weight management approaches varied widely among interviewees, reflecting diverse preferences and options. Lifestyle interventions were often at the core, and use of mental health care, OMs, and surgery, among other strategies, was also reported. All HCPs had experience prescribing OMs. Some noted OMs’ utility in individuals undergoing bariatric or metabolic surgery, but a larger-scale study on OM use among pre- and post-surgery patients could provide additional insight. Individuals in our study were largely aware of OMs (83%), especially younger cohorts (all aged 18–34 years versus 69% of those ≥65). In a 2022 survey of 917 adults with obesity, only 36% were aware of OMs [55]. The increased awareness in our study population relative to that reported in prior studies may reflect the growing media attention and popularity of OMs for weight loss. Interviewees in our study named a variety of OM information sources, such as social media, the Internet, HCPs, family, and friends. They emphasized the need for more educational resources about weight management approaches and OMs to better understand available options and make informed decisions.

The need to improve OM access was also evident. Less than one-third of individuals with overweight or obesity in our sample (28%) were using OMs at the time of interviews. However, all were eligible per the selection criteria based on self-reported data. While some may prefer not to use or cannot tolerate OMs, others cited an inability to afford or insufficient insurance coverage. Some patients may not have received prescriptions because of access considerations, noted by HCPs as a critical factor in deciding whether to prescribe OMs. Though our study participants were not paired, the same considerations may apply to the HCPs of the included individuals.

Interviewees also emphasized the importance of HCPs supporting patients navigating weight management, identifying it as an area for improvement. Despite all individuals in our study having an HCP accessible, only 58% had an HCP they found supportive in helping them lose weight. Individuals with higher BMI more frequently reported HCP involvement in weight management than those with lower BMI. Additionally, specialist referrals are not always accessible, with HCPs reporting that comorbidities, individual willingness, and insurance requirements influence these decisions. Discussions with HCPs were seen as critical sources of weight management guidance, but their execution was inconsistent and sometimes did not occur at all. Previous studies reported similar barriers to discussion, including lack of consultation time, prioritizing other health conditions, disconnect in HCP-patient relationships [36,[42], [43], [44],^69^], and limited scheduled follow-up care [36]. These observations suggest there is room for HCPs to be more proactive, engage patients in weight management dialogue sooner, and offer tangible solutions with clear direction. Specifically, HCPs could provide more comprehensive information on OMs to patients, especially on safety and tolerability, and allot time and resources for follow-up questions and support filling prescriptions.

Further, these interactions need not be limited to specialists alone. Over 90% of individuals in our study reported discussing weight with a PCP, the most common OM prescriber among those who received a prescription. Future research should explore strategies for PCPs to better support their patients’ weight management journeys and OM use, given their unique front-line positions.

This study's strengths include representation from a large proportion of non-White individuals with overweight or obesity and Spanish-speaking individuals, reflecting different cultural perspectives from communities highly impacted by obesity in the US [70]. Further, HCPs from a range of specialties offered unique perspectives on treating patients with obesity or overweight. Finally, we explored various topics, providing comprehensive insight into the experiences of individuals and HCPs considering weight management approaches.

## 5 Limitations

As for limitations, interview-based studies are subject to selection bias, self-reporting, and recall bias. This study was qualitative, with relatively small respondent samples, so it was infeasible to explore statistical correlations or associations between factors and outcomes. However, fundamental concepts of interest were coded and summarized, allowing for a broad interpretation of the most important concepts to individuals with overweight or obesity and HCPs, which may guide future work in this area.

As interviews were conducted virtually, participants in this study may represent those with greater technology access and digital literacy. Findings from our HCP interviews may not be generalizable to the broader HCP population, who may not all treat obesity as a medical condition or routinely prescribe OMs as our HCP interviewees did. In addition, the relatively small sample size of HCPs may not represent the full breadth of obesity treatment approaches that could be considered, including lifestyle counseling and behavioral interventions, which were not explored in-depth in this study given the emphasis on OM prescribing behaviors. A follow-up quantitative study with larger cohorts could further explore subthemes identified in our interviews and associations between participant characteristics and treatment experience, as well as expand on the perspectives from HCPs with less experience in obesity management.

## 6 Conclusion

In conclusion, this qualitative study demonstrated that overweight and obesity are complex and multifactorial conditions necessitating highly individualized treatment strategies. Education and proactive discussions with HCPs are vital to selecting weight management strategies best suited to an individual's needs, especially considering newer options such as OMs. Support and motivation are crucial for long-term success. In this context, expanding access to treatments, strengthening available resources, and enhancing HCP support represent key opportunities to better enable individuals to pursue and sustain weight management strategies and ultimately help improve their quality of life.

• Weight management requires individualized, proactive care.
• Missed opportunities in patient-provider communication concerning weight management persist.
• Sustained weight management success depends on support beyond treatment selection.

## Author contributions

Marie Louise Edwards, Urvi Desai, Ashley Holub, Alice Qu, Matthew Mattera, Natalia Ankerholz, Ellen Sears, and Noam Kirson contributed to study conception and design, collection and assembly of data, and data analysis and interpretation. Fatima Cody Stanford, Shraddha Shinde, Anthony Hasan, and Donna Mojdami contributed to study conception and design, data analysis, and interpretation. All authors reviewed and approved the final content of this manuscript.

## Ethical adherence and ethical review

The study was exempted from institutional review by Pearl Independent Review Board, Indianapolis, Indiana. Since this was not a clinical trial, there is no NCT number to report.

## Declaration of Artificial Intelligence (AI) and AI-assisted technologies

During the preparation of this work, the author(s) did not use AI.

## Source of funding

This study was funded by 10.13039/100004312Eli Lilly and Company.

## Declaration of competing interest

Fatima Cody Stanford has served as an advisor to Eli Lilly and Company, Novo Nordisk, Amgen, Currax, Boehringer Ingelheim, GoodRx, Doximity, Vida Health, Ilant Health, Calibrate, MelliCell, Sweetch, Empros Pharma, Clearmind Medicine, Pfizer, AbbVie, and AstraZeneca. Shraddha Shinde, Anthony Hasan, and Donna Mojdami are employees of Eli Lilly and Company. Donna Mojdami is a shareholder of Eli Lilly and Company. Urvi Desai, Matthew Mattera, Natalia Ankerholz, Ellen Sears, and Noam Kirson are employees of Analysis Group, Inc., a consulting company that has provided paid consulting services to Eli Lilly and Company, which funded the development and conduct of this study and manuscript. Alice Qu, Ashley Holub, and Marie Louise Edwards were employees of Analysis Group, Inc. at the time of writing but are not currently employed by the company.

## Data Availability

Due to the sensitive nature of the research, supporting data are not available to protect participant privacy.
