# Supplementary material for: One size does not fit all: Qualitative interviews with individuals with overweight or obesity and healthcare providers on navigating and supporting weight management
Source: Obes Pillars. 2026 Apr 1;18:100262. doi: 10.1016/j.obpill.2026.100262 (PMC13090608; doi:10.1016/j.obpill.2026.100262)
Supplement: Multimedia component 1 [file mmc1.docx]

**Appendix A. Supplementary materials**

*Supplemental methods: Analysis*

In the first phase of the hybrid deductive and inductive coding approach, we developed two a priori codebooks based on the semi-structured interview guides; one for interviews with individuals with overweight or obesity and one for interviews with HCPs. Four independent researchers (two for each participant group) reviewed an initial set of transcripts to identify commonly reported concepts and sub-concepts, and revised the codebooks to reflect the interview data. Following this, we estimated the Cohen’s kappa to assess inter-rater reliability for a subset of parallel coded transcripts (HCPs = >0.7; for individuals with overweight or obesity = >0.7). Discrepancies were adjudicated with the study team and the codebooks were finalized. We then used finalized codebooks to analyze all transcripts. We developed coding summary tables, which reported, for each concept and sub-concept, the number and proportion of reporting participants and associated number of supporting quotes.

In the second phase, we reviewed the concepts and sub-concepts identified in the interviews and developed narrative themes summarizing the key findings across both participant groups. Themes were refined and finalized based on discussions with the study team.

*Supplemental results: Recruitment and enrollment*

**Supplemental Figure 1** describes study recruitment and enrollment.

Individuals with overweight or obesity

A total of 122 individuals with overweight or obesity completed the screener and provided consent, and 13 were ineligible based on their screening responses. Among 109 eligible individuals, four were lost to follow-up (e.g., did not return requests to schedule), two were removed as they no longer wished to participate, and 63 were considered “over quotas”, who sought to enroll after the samples for their respective hard quotas (age, race/ethnicity) were already full. A total of 40 individuals completed the interviews.

HCPs

Among 40 HCPs screened, three were ineligible based on their screening responses (one of whom did not provide consent). Among 37 eligible HCPs, one HCP was removed before completion due to a technical issue with the interview recording and 11 were considered “over quotas”, who sought to enroll after the samples for their respective hard quotas (medical specialty, degree type) were already full. A total of 25 HCPs completed the interviews.

*Supplemental tables*

**Supplemental Table 1.** **Definition of successful weight management^1^**

|  | **Number of respondents** | **% of N** | **References^2^** |
| --- | --- | --- | --- |
| **Perspective of individuals with overweight or obesity** | | **N = 40** |  |
| **Definition of successful weight management** |  |  |  |
| Weight loss | 14 | 35% | 16 |
| Keeping weight off | 9 | 23% | 11 |
| Becoming healthier or controlling comorbidities not just weight loss | 9 | 23% | 10 |
| Self-control or self-discipline | 6 | 15% | 9 |
| Getting back to normal eating and exercising | 4 | 10% | 4 |
| Implementing lifestyle changes | 4 | 10% | 4 |
| **Perspective of HCPs** | | **N = 25** |  |
| **Definition of successful weight management** |  |  |  |
| Patient loses weight | 20 | 80% | 29 |
| 1-10% of bodyweight | 10 | 40% | 12 |
| >10% of bodyweight | 6 | 24% | 7 |
| Patient satisfaction | 11 | 44% | 11 |
| Adherence to the strategy | 6 | 24% | 7 |
| Improvement in comorbidities | 6 | 24% | 9 |
| Not gaining further weight | 3 | 12% | 3 |

**Abbreviation**: HCP=Healthcare provider

**Notes**:

Response options reported by at least three participants are shown.

Refers to number of times mentioned by respondents

**Supplemental Table 2. Perspective of HCPs on referrals to specialists or surgery^1^**

|  | **Number of respondents** | **% of N** | **References^2^** |
| --- | --- | --- | --- |
| **Perspective of HCPs** | | **N = 25** |  |
| **Person who primarily initiates discussions about referrals or bariatric surgery** |  |  |  |
| HCP | 14 | 56% | 14 |
| Patient | 6 | 24% | 8 |
| A mix | 4 | 16% | 4 |
| **Type of specialist to whom patients are referred** |  |  |  |
| Nutritionist | 17 | 68% | 31 |
| Mental health provider | 17 | 68% | 27 |
| Bariatric surgeon | 14 | 56% | 17 |
| Endocrinologist | 12 | 48% | 14 |
| Other (e.g., neurologist, gastroenterologist, sleep specialist) | 8 | 32% | 12 |
| Physical therapist | 5 | 20% | 5 |
| Exercise physiologist | 5 | 20% | 6 |
| Cardiologist | 4 | 16% | 5 |
| Well-established diet centers | 3 | 12% | 4 |
| **Factors that influence specialist referrals** |  |  |  |
| Comorbidities | 10 | 40% | 13 |
| Patient willingness | 6 | 24% | 7 |
| Insurance requirements | 5 | 20% | 5 |
| Disordered eating | 4 | 16% | 5 |
| Gap in care | 3 | 12% | 3 |
| **Type of surgical intervention** |  |  |  |
| Sleeve gastrectomy | 8 | 32% | 8 |
| Gastric bypass | 5 | 20% | 5 |
| Endoscopic procedure | 4 | 16% | 5 |
| **Factors that influence surgery referrals** |  |  |  |
| Severity of overweight or obesity | 8 | 32% | 11 |
| Tolerability - other treatment options failed | 6 | 24% | 8 |
| Patient has many comorbidities | 3 | 12% | 6 |
| Patient motivation | 3 | 12% | 4 |
| Patient characteristics | 3 | 12% | 4 |

**Abbreviation**: HCP=Healthcare provider

**Notes**:

Response options reported by at least three participants are shown.

Refers to number of times mentioned by respondents

**Supplemental Table 3. Weight management discussions^1^**

|  | **Number of respondents** | **% of N** | **References^2^** |
| --- | --- | --- | --- |
| **Perspective of individuals with overweight or obesity** | | **N = 40** |  |
| **Initiator of the discussion** |  |  |  |
| HCP | 20 | 50% | 28 |
| Patient | 13 | 33% | 17 |
| **Discussion of weight management** |  |  |  |
| Extent of discussion |  |  |  |
| Discussion led to referral | 20 | 50% | 46 |
| Dietitian or nutritionist | 15 | 38% | 21 |
| Bariatric surgeon | 5 | 13% | 7 |
| Therapist | 5 | 13% | 6 |
| Discussion was related to other health conditions | 17 | 43% | 31 |
| Discussion was about OMs | 16 | 40% | 25 |
| Led to implementation or actions of the patient | 16 | 40% | 23 |
| Changes in diet or exercise or habits | 13 | 33% | 20 |
| Seeing a recommended HCP (non-referral) | 3 | 8% | 3 |
| Discussion did not lead to further actions | 13 | 33% | 19 |
| Provided lifestyle intervention advice | 6 | 15% | 11 |
| Discussion was about bariatric metabolic surgery | 5 | 13% | 8 |
| Discussion not at every visit | 5 | 13% | 7 |
| **Perceived reason for lack of discussion on weight management** |  |  |  |
| HCP focused on health conditions other than weight | 8 | 20% | 11 |
| HCP didn't bring it up | 4 | 10% | 12 |
| Patient has not sought weight management help | 3 | 8% | 5 |
| **Information shared by HCP when OMs were prescribed** |  |  |  |
| Features of OMs | 11 | 28% | 25 |
| Benefits and effectiveness | 8 | 20% | 10 |
| Safety and adverse events | 7 | 18% | 9 |
| Administration and dosage | 5 | 13% | 5 |
| Costs and insurance coverage | 3 | 8% | 3 |
| **Perspective of HCPs** | | **N = 25** |  |
| **Person who primarily initiates discussions about weight management strategies** |  |  |  |
| A mix | 12 | 50% | 15 |
| HCP | 6 | 24% | 7 |
| Patient | 4 | 16% | 7 |
| **Point in care when weight management strategies are discussed** |  |  |  |
| Patient has or is at risk of developing comorbidities | 19 | 76% | 37 |
| On the first visit | 13 | 52% | 17 |
| Every visit | 12 | 48% | 18 |
| Timing dependent on severity of overweight or obesity | 9 | 36% | 10 |
| The patient requests information | 8 | 32% | 16 |
| Second or third visit | 7 | 28% | 10 |
| Other timing consideration (i.e., patient event) | 6 | 24% | 7 |
| After patient-provider trust is developed | 3 | 12% | 3 |
| **Factors that prompt weight management discussions** |  |  |  |
| Comorbidities | 21 | 84% | 41 |
| Patient motivation | 16 | 64% | 28 |
| Patient having trouble losing weight | 8 | 32% | 9 |
| Potential benefit to patients | 6 | 24% | 9 |
| Drug advertising | 4 | 16% | 5 |
| **Factors that help make discussing weight management comfortable** |  |  |  |
| Patients are receptive | 17 | 68% | 35 |
| HCP experience having these discussions | 14 | 56% | 36 |
| Discuss how overweight or obesity is impacting other medical conditions | 12 | 48% | 29 |
| Shared decision-making | 12 | 48% | 18 |
| Health literacy or patient knowledge | 12 | 48% | 21 |
| Patient willingness to pursue treatment | 8 | 32% | 8 |
| Non-judgmental approach | 5 | 20% | 6 |
| Patient characteristics | 5 | 20% | 7 |
| **Information typically shared with patients regarding OMs** |  |  |  |
| Features of OMs | 25 | 100% | 99 |
| Side effects | 22 | 88% | 29 |
| Dosage | 13 | 52% | 14 |
| Length of use | 13 | 52% | 15 |
| Efficacy | 11 | 44% | 13 |
| Route of administration | 11 | 44% | 13 |
| Mechanism of action | 10 | 40% | 11 |
| Insurance or cost | 16 | 64% | 16 |
| Weight loss expectations | 15 | 60% | 22 |
| Frequency of healthcare visits needed | 10 | 40% | 11 |
| Contraindication | 3 | 12% | 4 |
| Impact on comorbidities | 3 | 12% | 4 |

**Abbreviations**: HCP=healthcare provider; OMs=obesity medications

**Notes**:

Response options reported by at least three participants are shown.

Refers to number of times mentioned by respondents

**Supplemental Table 4. Motivation and support for weight management^1^**

|  | **Number of respondents** | **% of N** | **References^2^** |
| --- | --- | --- | --- |
| **Perspective of individuals with overweight or obesity** | | **N = 40** |  |
| **Factors that have been helpful for weight management** |  |  |  |
| Support from friends or family | 22 | 55% | 32 |
| Accountability buddy or peer support group | 5 | 13% | 6 |
| Support from HCP | 5 | 13% | 9 |
| Being able to afford treatments or programs | 4 | 10% | 4 |
| Self-discipline | 3 | 8% | 5 |
| Better lifestyle practices | 3 | 8% | 4 |
| **Current role and involvement of HCPs** |  |  |  |
| HCP involved with weight management | 23 | 58% | 77 |
| HCP is supportive towards weight loss | 15 | 38% | 20 |
| HCP comes up with weight management strategies | 12 | 30% | 23 |
| HCP explains and provides sufficient information | 7 | 18% | 9 |
| HCP provides personalized patient-centered care | 7 | 18% | 9 |
| HCP follows up on weight loss progress | 4 | 10% | 6 |
| Patient receives continuum of care | 4 | 10% | 5 |
| Holistic approach | 3 | 8% | 3 |
| HCP not involved or has limited involvement with weight management | 23 | 58% | 61 |
| HCP acknowledges weight issue but doesn't provide solutions | 13 | 33% | 25 |
| HCP currently focused on comorbidities or health conditions other than overweight obesity | 8 | 20% | 11 |
| HCP doesn't acknowledge weight issue nor provide solutions | 6 | 15% | 10 |
| HCP unsupportive of weight loss | 5 | 13% | 6 |
| Lack of continuum of care | 4 | 10% | 5 |
| **Perspective of HCPs** | | **N = 25** |  |
| **How patients are motivated to stay on track in their weight management journey** |  |  |  |
| Show improvements the patient made | 12 | 48% | 13 |
| Regular appointments | 11 | 44% | 11 |
| Positive reinforcement | 9 | 36% | 11 |
| Discussing comorbidities | 6 | 24% | 10 |
| Setting realistic goals | 3 | 12% | 3 |
| Using apps to track weight or diet | 3 | 12% | 3 |
| **How patients are motivated to take OMs** |  |  |  |
| Setting realistic goals | 5 | 20% | 7 |
| Show improvements the patient made | 3 | 12% | 3 |
| Scheduling frequent follow-up visits | 3 | 12% | 5 |
| **How success with a weight management strategy is monitored** |  |  |  |
| Weight numbers | 11 | 44% | 12 |
| Lab values | 9 | 36% | 9 |
| Patient's satisfaction | 6 | 24% | 6 |
| Quality of life | 4 | 16% | 4 |
| Regular appointments or check-ins on weight strategy | 4 | 16% | 5 |
| Comorbidity outcomes | 3 | 12% | 3 |

**Abbreviations**: HCP=healthcare provider; OMs=obesity medications

**Notes**:

Response options reported by at least three participants are shown.

Refers to number of times mentioned by respondents

**Supplemental Table 5. Room for improvement to support weight management^1^**

|  | **Number of respondents** | **% of N** | **References^2^** |
| --- | --- | --- | --- |
| **Perspective of individuals with overweight or obesity** | | **N = 40** |  |
| **Room for improvement for discussion with HCPs** |  |  |  |
| **Expected role and involvement of HCP** |  |  |  |
| HCP provides options or solutions for weight management | 13 | 33% | 21 |
| HCP provides more support for weight management | 12 | 30% | 15 |
| HCP initiates discussion on weight management | 6 | 15% | 10 |
| Personalized patient-centered care | 5 | 13% | 6 |
| HCP holds patient accountable | 4 | 10% | 5 |
| Patient receives continuum of care | 3 | 8% | 3 |
| **Support from HCPs or healthcare system that would be helpful** |  |  |  |
| Recommendations from HCP | 7 | 18% | 11 |
| Clinical support system | 6 | 15% | 8 |
| More consultation time | 5 | 13% | 5 |
| Referrals from HCP to other specialists | 4 | 10% | 5 |
| Therapy | 3 | 8% | 4 |
| More clinical approach | 3 | 8% | 3 |
| **Room for improvement for education** |  |  |  |
| **Factors that would be helpful for weight management related to support and education** |  |  |  |
| Social support | 24 | 60% | 39 |
| Accountability buddy or peer support group | 15 | 38% | 21 |
| Support from family and friends | 8 | 20% | 10 |
| Workout partner | 6 | 15% | 7 |
| More information or education | 4 | 10% | 5 |
| Education on different weight management methods | 4 | 10% | 5 |
| **Information interviewees would like to know about OMs** |  |  |  |
| Features of OMs | 24 | 60% | 62 |
| Safety and adverse events | 19 | 48% | 28 |
| Benefits and effectiveness | 12 | 30% | 16 |
| Clinical background and research | 6 | 15% | 8 |
| Long-term effects | 5 | 13% | 6 |
| Cost and insurance coverage | 5 | 13% | 5 |
| **Room for improvement for OM access** |  |  |  |
| **Factors that would be helpful for weight management related to insurance and access** |  |  |  |
| Insurance coverage support and affordable solutions | 11 | 28% | 17 |
| Access to resources | 5 | 13% | 5 |
| **Beliefs about treatment accessibility** |  |  |  |
| Healthcare is not accessible enough | 13 | 33% | 25 |
| Lack of insurance coverage of options | 4 | 10% | 4 |
| Treatments are not affordable | 4 | 10% | 8 |
| Lack of patient-centered care | 4 | 10% | 5 |
| Unmet need for more information and education | 3 | 8% | 4 |
| Insurance coverage is important | 13 | 33% | 24 |
| **Reason patient did not fill an OM prescription** |  |  |  |
| Cost or insurance did not cover it | 4 | 10% | 10 |
| **Perspective of HCPs** | | **N = 25** |  |
| **Room for improvement for discussion with HCPs** |  |  |  |
| **Barriers to discussing weight management** |  |  |  |
| Patient is not concerned with overweight or obesity | 15 | 60% | 23 |
| Prioritize treatment of comorbidities | 9 | 36% | 10 |
| Patient past negative experience | 8 | 32% | 9 |
| Societal pressures or stigma | 7 | 28% | 7 |
| HCP time | 6 | 24% | 10 |
| Patient knowledge | 5 | 20% | 6 |
| Patient demographics | 5 | 20% | 10 |
| Patient mental health | 4 | 16% | 5 |
| **Room for improvement for education** |  |  |  |
| Obesity is a complex disease that needs more resources and research | 11 | 44% | 13 |
| Education is important for HCPs and patients, and more is needed | 7 | 28% | 7 |
| Patient and HCP awareness | 3 | 12% | 3 |
| Lots of potential for new treatment innovations, more HCP education on treatments needed | 2 | 8% | 2 |
| **Room for improvement for OM access** |  |  |  |
| Access to medication is important and needs to be improved | 8 | 32% | 11 |
| Better medication options needed | 4 | 16% | 4 |
| Needs to be more access to OMs | 4 | 16% | 4 |
| **Reason patients do not fill their prescriptions** |  |  |  |
| Financial factors | 14 | 56% | 14 |
| Insurance or copay card | 7 | 28% | 7 |
| Medication shortage | 3 | 12% | 3 |
| Patient not motivated | 3 | 12% | 3 |

**Abbreviations**: HCP=healthcare provider; OMs=obesity medications

**Notes**:

Response options reported by at least three participants are shown.

Refers to number of times mentioned by respondents

*Supplemental figure*

**Supplemental Figure 1. Individuals with overweight or obesity (A) and HCP (B) recruitment and enrollment**

**A B**


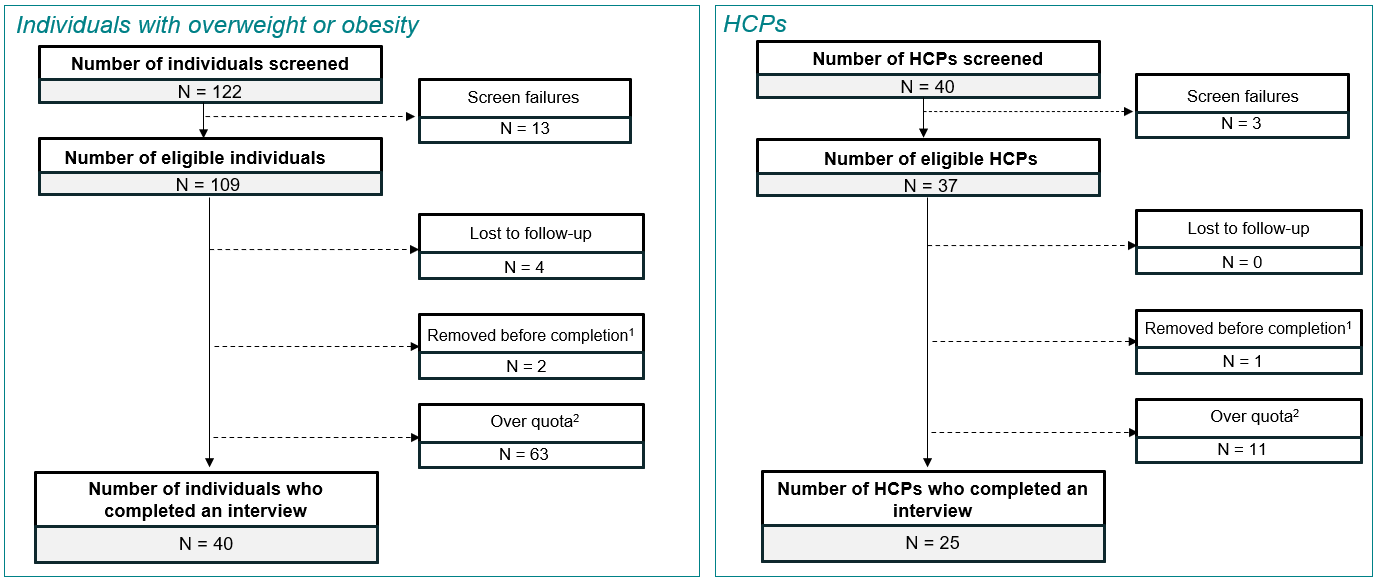


**Abbreviation:** HCP=healthcare provider

**Notes**:

1. Two individuals were removed before completion due to no longer wanting to participate in the study and 1 HCP was removed before completion due to an issue with the interview recording.

2. Overquotas are participants who sought to enroll after the samples for their respective hard quotas (e.g., age group, HCP specialty) were already full.
